# Structures and processes necessary for providing effective home treatment to severely mentally ill persons: a naturalistic study

**DOI:** 10.1186/s12888-016-0945-z

**Published:** 2016-07-15

**Authors:** E. Bauer, K. Kleine-Budde, C. Stegbauer, P. Kaufmann-Kolle, K. Goetz, B. Bestmann, J. Szecsenyi, A. Bramesfeld

**Affiliations:** AQUA – Institute for Applied Quality Improvement and Research in Health Care, Maschmuehlenweg 8-10, 37073 Goettingen, Germany; Department of General Practice and Health Services Research, University Hospital Heidelberg, Voßstr. 2, 69115 Heidelberg, Germany; Institute of Family Medicine, University Hospital Schleswig-Holstein, Campus Luebeck, Luebeck, Ratzeburger Allee 160 / Haus 50, 23538 Luebeck, Germany; Scientific Institute of TK for Benefit and Efficiency in Health Care (WINEG), Bramfelder Str. 140, 22305 Hamburg, Germany; Department Epidemiology, Social Medicine and Health System Research, Hannover Medical School, Carl-Neuberg-Str. 1, 30625 Hannover, Germany

**Keywords:** Quality assessment, Mental health services, Integrated care, Claims data, Health services research, Quality of care

## Abstract

**Background:**

Home treatment for severely mentally ill persons is becoming increasingly popular. This research aims to identify structures and processes in home treatment that impact on patient-related outcomes.

**Methods:**

We analysed 17 networks that provide home treatment to severely mentally ill persons using a naturalistic approach. The networks were similar with regard to central components of home treatment such as case management, 24 h crisis hotline and home visits, but differed in all other aspects such as the multidisciplinary teams, time spent with patients, etc. To determine treatment outcome, patients’ psychosocial functioning was measured using the Health of the Nation Outcome Scales (HoNOS). Structures and processes were assessed using claims data and questionnaires answered by the different networks. Primary outcome was highlighted by the change in HoNOS scores from the start of home treatment compared with 6 months later. We sought to explain this outcome through patient and network characteristics using regression analysis. Data on 3,567 patients was available.

**Results:**

On average, psychosocial functioning improved by 0.84 across networks between t0 and t1. There were more similarities than differences between the networks with regard to the structures and processes that we tested. A univariate regression analysis found staff’s prior experience in mental health care and the effort that they invested in their work correlated positively with patient outcome. This needs to be interpreted under considering that univariate analysis does not show causal relationship. A high case load per case manager, increased and longer patient contact and more family intervention were correlated with worse patient outcome, probably indicating that sicker patients receive more care and intervention.

**Conclusion:**

Home treatment networks succeed in delivering care tailored to the needs of patients. In order to improve the quality of care in home treatment, this study suggests employing experienced staff who is ready to invest more effort in their patients. Further research needs to consider a longer follow-up time.

**Electronic supplementary material:**

The online version of this article (doi:10.1186/s12888-016-0945-z) contains supplementary material, which is available to authorized users.

## Background

During the last few years, an increasing number of care models have been established worldwide which offer home treatment, case management and multidisciplinary care to persons who are severely mentally ill. Available evidence suggests that home treatment provided to the severely mentally ill by multidisciplinary psychosocial intervention teams has the potential to be effective with regard to suicide prevention, promotion of patient satisfaction and the need for inpatient treatment [1–3].

Also in Germany, models that offer some form of home treatment within the framework of integrated care are becoming more and more popular [4]. Prior studies have shown that the effectiveness of home treatment programmes is related to the structures and processes of these programmes [5]. Factors revealed to be relevant to patient outcomes in home treatment include the caseload per case manager, regular and frequent home visits, accountability of home treatment programmes for medical and social issues, multidisciplinary teams that include psychiatrists [6], and burnout levels of staff [7]. Evidence is still scarce as to what extent these and other structures and processes in home treatment contribute to its effectiveness. The research presented here aims to identify structures and processes in home treatment of severely mentally ill persons that probably impact on patient-related outcomes and that are beyond the central components of the intervention such as the existence of case management, availability of 24 h crisis hotlines and home visits.

## Methods

The structures and processes of 17 regional home treatment networks that provide similar, but nonetheless, individually different types of home treatment were analysed. These regional care networks called the German Network for Mental Health (*das deutsche NetzWerk psychische Gesundheit* (NWpG)) were initialized in 2009 by the Techniker Krankenkasse (TK), one of the largest statutory health insurance companies in Germany, with more than nine million insured persons. By providing home treatment as well as case management to the severely mentally ill, the NWpG’s aim was to reduce the need for mental health inpatient care. Only patients who are insured with the TK and who fulfil certain criteria in respect to their course of illness are eligible for being treated on this programme. The TK selects patients for the programme through an algorithm applied to health insurance claims data [8]. This algorithm filters those patients carrying the highest risk for hospitalization due to mental illness. The risk for hospitalization is assessed by predictive modelling using among others data on use of inpatient care for mental disorders and psychotropic medication as well as receiving the diagnosis of mental disease in the past year(s). The TK contacts patients that score lies above a certain threshold in predictive modelling and proposes them to enrol in the programme. In 2013, NWpG networks existed in 11 of 16 federal states with a focus in northwest Germany; in 2015 they are present in more than 25 regions. Except for one network all networks operate in urban and rural areas. The time that networks were existing varied between 19 months prior to data collection (October 2013) and 4 years. In 2012 there were more than 9,000 TK insured persons that had signed up for the programme since it started in 2009.

All networks provide similar core services that include: home treatment and case management, sociotherapy, psychoeducation, a 24 h crisis hotline and crisis intervention apartments. Apart from these available services that are standard, the networks are free to choose how they organize themselves, e.g., which professionals they employ (nurses, social workers, psychologists, patient experts), who steers the case management (psychiatrist or social worker), with what other local services they cooperate and to whom they propose specific interventions such as sociotherapy.

### Study design and data basis

For this comparison, network data was derived from three different sources:

1. Routine assessments of psychosocial functioning
2. Questionnaires on structures and processes applied to the networks
3. Health care claims data for the patients enrolled on the NWpGs.
  1. Routine assessment of psychosocial functioning was performed under contractual obligation at admission and every 6 months thereafter. The assessment was done by the patient’s case manager, who was unaware of the purpose of this study. Psychosocial functioning was assessed by means of the German version of the Health of the Nation Outcome Scales (HoNOS) [9]. It is a third party assessment tool, with scores ranging from 0 to 48 points; thereby higher scores indicate more impairment in psychosocial functioning. HoNOS data was linked to the patient’s claims data via pseudonymized patient IDs. Relative change in the HoNOS over time was the outcome.
  2. A questionnaire on non-standardized structures and processes of the networks was completed by the network managers (for network questionnaire see Additional file 1). Thereby, overall characteristics of the networks were assessed, such as the number of staff, the staff / patient ratio, the staff professions and outside cooperation partners of the networks. In addition, a further questionnaire was completed by the staff involved in direct patient care (for staff questionnaire see Additional file 2). It addressed issues like time and frequency of home visits, time spent with patients but also job satisfaction and psychosocial stress in the work place. This questionnaire referred generally to the status of the last 3 months prior to completion of the questionnaire. Both questionnaires were completed between November 2013 and January 2014. We developed the questionnaires based on a systematic literature review and a Delphi like discussion process with each network on the structures and processes they believed to be relevant for the quality of care that the network provided [10].
  3. Health care claims data was finally used to include information on patient demographics, use of inpatient treatment, medication, somatic and psychiatric diagnoses and use of outpatient care. The data was available for each patient starting from the year prior to enrolment in the NWpGs up until June 2013. Since not all the patients enrolled at the same time, patient information was restructured according to the individual date of enrolment (t0). Patients were anonymized by the TK making it impossible to trace their identity.

For data analysis the results of the staff questionnaires and also patient information through claims data were aggregated at network level. Thus, they became one of the “characteristics” of the networks. Linking this data to the respective networks was possible via network ID.

In summary, the resulting data collection included information on the following:

- Patient outcomes (HoNOS).
- Patient characteristics, such as age, sex, somatic comorbidity summarized by the Charlson Index [11], mental comorbidity summarized in four groups: 1) schizophrenic and related disorders (ICD 10 F20-F29); 2) mood and affective disorders (ICD-10 F30-F39); 3) neurotic, stress-related and somatoform disorders (ICD-10 F40-F48); and 4) all other ICD-10 F-diagnoses.
- Non-standardized structures and processes, such as number of patients per case manager, time case managers spend with patients, number of patients per network, cooperation agreements with other care providers, and staff professions. Structures and processes assessed are presented in Table 2.
- Staff characteristics for each network, such as demographics, education, years spent in mental health care and in their particular job, amount of contact with enlisted patients over a period of 3 months, job satisfaction and stress in the work place (Effort-Reward-Imbalance-Questionnaire [12, 13]).

Network names are not published. They are numbered consecutively in a descending order according to the number of enrolled patients from 1 to 17. The ethics commission of the State Medical Chamber of Lower Saxony (*Ethikkommission bei der Ärztekammer Niedersachsen*) ruled that an ethics approval was not required since patients were not directly involved in the study.

### Patients

All patients aged 18 years or older who were treated from 2009 to July 2013 by one of the 17 networks for more than 6 months were eligible subjects to this study (n = 7,243). Of these patients about half had to be excluded due to incomplete HoNOS data: 2,174 patients lacked a HoNOS score at either t0 or t1; most likely due to delays in data transfer from the networks to the health insurance and further on to the external managed data base where data was stored. In addition, 1,502 patients had to be excluded because of too many missings in one of their HoNOS scores. 3,567 patients remained; who’s HoNOS scores at both t0 and t1 were complete and could be included in the study. Patients included in the study and those excluded, did not differ significantly by gender or diagnoses. Patients included were slightly older (46.1 years) as compared to those excluded (45.3 years).

### Outcome

The intended outcome of the analyses was the clinical improvement of patients over time, as represented by the relative change in the HoNOS between t0 and t1.

### Statistical analysis

To determine whether and which network structures and processes induce a more positive patient- related improvement, a univariate linear regression analysis and multilevel regression was applied [14]. In both analyses patients’ improvement in the HoNOS is presumed to originate from structures and processes of the network to which the patient is associated. With the univariate linear regression each independent variable was measured individually against the dependent variable.

In the multi-level analysis the relative change in the HoNOS between t0 and t1 served as the patient dependent variable that was explained by variables such as age, sex and comorbidity at patient level (1^st^ level) and network characteristics at network level (2^nd^ level). For all statistical procedures SPSS 21 advanced statistics were used. Statistical significance was defined as *p* < .05 for all analyses.

## Results

### Patient characteristics

With the exception of the HoNOS, all displayed variables relating to patients were only slightly different (see Table 1). The mean age of the patients per network ranged between 44.8 and 50.7 years, with an average age of 46.1 years. Similar to the distribution of patient’s age among networks and the percentage of women, psychiatric diagnosis groups according to ICD-10 and the Charlson Index indicate that there were very few differences between the patient populations of the networks. As can be seen in Table 1 most patients were carrying more than one diagnosis of mental disorder. This shows that networks served predominantly patients with complex mental disorders. It should however, be noted that the HoNOS was not equally distributed across networks. While its mean value remained at a score of 12.28, the network specific score ranged from 9.07 to 18.21. This indicates that the patients in some networks were on average twice as sick as in other networks. Further calculations of confidence intervals confirmed these HoNOS differences as being statistically significant.

**Table 1:**
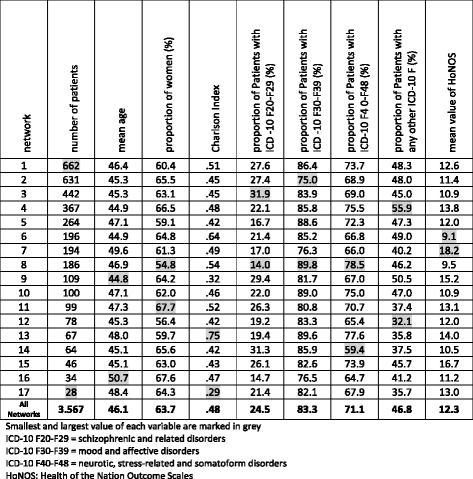
Characteristics of patients enrolled in home-treatment networks at t0

### Psychosocial functioning outcome

**Fig. 1:**
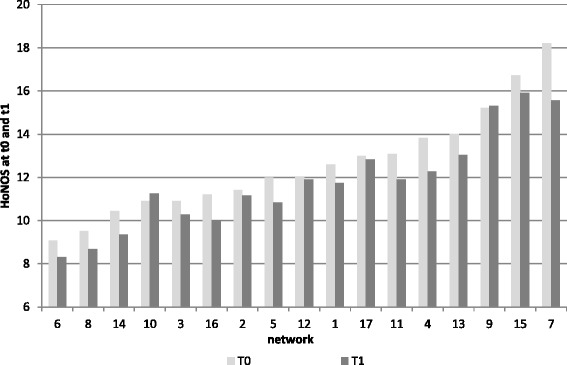
Average functional status per enlisted patient measured by Health of the Nation Outcome Scales (HoNOS) among networks at t0 and t1

Figure 1 presents the average HoNOS score per patient at t0 and t1 for each network. Since the measurement at t0 was conducted around the time of enrolment and the measurement at t1 about 6 months later, the discrepancy between both columns per network showed whether the mental condition of the patient had improved. On average, a statistically significant improvement of 0.84 (95 % confidence interval from −1.04 to -.65) on the HoNOS was noticed.

### Structures and processes

All tested network structures and processes, their median and the range across networks together with their relationship to the HoNOS are presented in Table 2. While some structures and processes differ considerably between networks (such as the proportion of patients receiving psychoeducation), the number of years of experience the staff have in mental health care and the number of patients per case manager are somewhat similar. To identify possible correlations between a patient’s improvement and the network characteristics, the relative change in the HoNOS between t0 and t1 as a dependent variable was modelled separately for testing each of the 23 identified network structures and processes for its relation to patient outcome. This represented a better patient outcome being positively correlated (negative beta coefficient) with a higher caseload per case manager, with staff investing more effort in their work and with them having more years of experience in mental health care. In addition, the following structures and processes correlated negatively (positive beta coefficient) with patient outcomes: a high number of home visits, a high share of patients whose family was contacted frequently, long-lasting face-to-face contact, increased staff job satisfaction and a high number of patients per network. It should be noted that the goodness of fit measured by R^2^ and the beta coefficient were close to zero, indicating only a weak correlation. Therefore, the results should be interpreted with caution.

**Table 2:** Structures and processes available in networks and their relationship to the patients’ improvement in Health of the Nation Outcome Scales (HoNOS) as determined via linear regression

	network description			regression statistics		
network characteristics	median	minimal value	maximal value	*R* ^2^	beta	*p*-value
Variables that characterise network structures						
Number of patients in a network	299	84	5487	0.00	0.04	0.03
Number of patients per case manager (measured by full-time equivalent)	40	19.67	69	0.00	−0.07	<0.01
Multiprofessionality of treatment team (number of different professions within the team of case managers)	3.5	1	7	0.00	−0.02	0.26
Number of organizations having a contract for cooperating with the network	3	1	6	0.00	0.00	0.97
Average number of all contacts with patients during the last 12 months	26.87	2.83	51.6	0.00	−0.03	0.06
Variables that characterise the staff						
Years of staff’s experience in mental health care	15.13	5.59	21.86	0.00	−0.04	0.03
Share of working hours spend with patient care during an average week	41 %	22 %	69 %	0.00	0.01	0.48
Average number of hours spent on home visits to patients during the last month	1.54	0.35	3.44	0.00	−0.01	0.63
Average number of training hours received by case managers during the last 12 months	46.33	5	106.5	0.00	0.00	0.89
Variables that characterise the staff’s work place satisfaction						
Staff’s work place satisfaction (range 1 = very dissatisfied to 7 = very satisfied)	5.32	4.25	6.11	0.00	0.03	0.04
Staff’s satisfaction with income (range 1 = very dissatisfied to 7 = very satisfied)	3.14	2	5.71	0.00	−0.02	0.20
ERI score – effort (range 3 to 12, high ratings point to higher efforts)	8.63	6	10.5	0.00	−0.05	0.01
ERI score – reward (range 7 to 28, low ratings point to lower rewards)	18	14.75	23.67	0.00	−0.01	0.65
ERI score – overcommitment (range 6 to 24, high ratings point to higher overcommitment)	13	11.33	16	0.00	0.00	0.97
Variables that characterise staff’s processes (contact variables)						
Number of face-to-face contacts per patient during the last month	1.3	1.13	1.75	0.00	0.03	0.05
Average number of hours of face-to-face contacts with patients during the last month	1.76	1.21	2.62	0.00	0.04	0.04
Average number of home visits to patients during the last month	1	0.29	1.79	0.00	0.05	<0.01
Share of patients, whose family members were contacted by the network during the last month	16 %	8 %	51 %	0.00	0.04	0.03
Variables that characterise staff’s processes (service variables)						
Share of patients receiving psycho-education during the last three months	27 %	8 %	68 %	0.00	−0.02	0.21
Share of patients receiving psychotherapy during the last three months	10 %	0 %	50 %	0.00	0.00	0.78
Share of patients receiving sociotherapy during the last three months	11 %	0 %	89 %	0.00	−0.03	0.11
Share of patients to which a treatment plan was handed during the last 12 months	33 %	0 %	93 %	0.00	−0.02	0.17
Share of patients receiving a case review during the last three months	41 %	5 %	93 %	0.00	0.02	0.31

The median depicts the value of the median network. Minimum and maximum values refer to the value of those networks with the lowest and highest figure. For each separate univariate linear regression the patients’ improvement as the dependent variable was explained by one of the listed network characteristic. Each row therefore describes one model

Beta-coefficient represents the influence of the variable on the outcome, a negative beta-coefficient implies a higher improvement e.g.: beta = -0.07 – more patients per case manager is correlated with higher improvement in patient outcome

A positive beta-coefficient implies a worsening of the outcome. Beta = 0.05 – A higher average number of home visits to patients within one month is correlated with worsening of the patients outcome

When considering processes other than home visits and face-to-face contact, statistical non-significant trends could be seen for a higher share of patients receiving sociotherapy, having a treatment plan and psychoeducation. These correlated positively with a better outcome. Also, staff being satisfied with their income showed a trend towards a positive outcome.

In an attempt to analyse in more detail the relationship between a patient’s improvement and characteristics of the patients (1^st^ level) and also of the networks (2^nd^ level), a multilevel analysis was conducted. However, applying structures and processes in a multilevel analysis did not succeed in explaining patient outcomes. Despite various attempts and by adding 2^nd^ level variables one by one, the models did not converge. At the first level of analysis, only a patient’s age related to an improvement in HoNOS scores at t1; at the second level, none of the included factors led to noteworthy results. Beyond the possibility that structures and processes were not related to the outcome, the comparatively small differences between the networks in what concerns their structures, processes and outcomes may explain the non-converging of the models (intra-class correlation of the unconditional model was .03 %).

## Discussion

This study focuses on the impact of structures and processes, other than the central standardised components of the home treatment model under investigation (case management, 24 h crisis hotline and home visits), on patient outcome. Using a naturalistic approach, 17 home treatment networks were studied in routine care.

In essence, beyond certain characteristics of the staff (experience in mental health care and the amount of effort put into their work), none of the non-standardised components in home treatment seemed to matter much in respect to patient outcome after 6 months of treatment. Probably the standardised components of the home treatment networks, in particular the fact that home treatment was provided at all, have so much more effect during these first months, that it is of minor importance as to whether the networks contract with a lot of outside service providers, or whether their personnel spends a greater share of its daily working time with patients. However, some of the non-standardised interventions, such as psychoeducation, providing a treatment plan and sociotherapy, which is a specific intervention for supporting the participation in social and professional life, showed a trend, albeit non-significant, towards being correlated with better patient outcome. If conclusions should be drawn from these results, it should be that it is probably better to offer these interventions, than not to have them at all.

The fact that the following were associated with less improvement to patient outcome: more patients per case manager, a higher number of home visits per patient, a higher share of patients whose family was contacted, more face-to-face contact and also more contact in general to patients, should be interpreted by considering that the applied regression analysis does not show causal relation. Therefore, these results probably show that sicker patients are receiving more attention and interventions by staff, e.g., more home visits, more family intervention, and more contact and care in general. Thus, the home treatment networks do in fact what they are supposed to do. This is, given the fact that it is a naturalistic study, a promising result.

If we had to make cautious recommendations on the basis of this research, it would be that home treatment overall, as a visiting mental health service might be recommendable because also in a routine care it succeeds in providing a higher intensity of care to those that are sicker and after 6 months of treatment patients are usually better off in respect of psychosocial functioning. For better patient outcomes it would be advisable to have staff in networks who are highly experienced in mental health care and who are ready to put a lot of effort into their work. Moreover, it would probably also be better if staff were satisfied with their income (a slight, however non-significant trend towards this finding). Furthermore, when considering other interventions beyond the mere home visits, it could be useful if networks provide sociotherapy, psychoeducation and treatment plans. Evidence from this study is not strong enough to state the absolute necessity of these three interventions. However, these interventions are also considered part of the treatment model by other home treatment models, such as the Functional Assertive Community Treatment (“FACT”), a Dutch version of assertive community treatment [15, 16]. It is recommended by treatment guidelines [17–20]. Furthermore, sociotherapy, psychoeducation and providing treatment plans (implying that a treatment plan is in fact present and developed in cooperation with the patient) are all measures to assist patients. This highlights their potential to contribute to better patient outcomes [17].

### Strengths and limitations

It should be considered as one of the strengths of this study that it is a naturalistic study exploring the effect of components of home treatment in a real world setting. A further strength relates to the outcome that considers the psychosocial functioning of the patient instead of using the use of inpatient services as an outcome only. The use of psychiatric inpatient services that is often taken as a surrogate for the wellbeing of patients is not only determined by the health of the patients but also by the availability of in- and outpatient services in a region [21].

Overall, the psychosocial status of the patients improved across networks, which was measured by an average decrease in the HoNOS of .84 points. This improvement is in line with findings of controlled studies indicating the general effectiveness of home treatment [22]. However, from a clinical point of view, this change in the HoNOS cannot be considered as being particularly strong [23]. Clinically, a change in the HoNOS of 4 points during a whole year is regarded as minimal improvement.

Unfortunately, we could include fewer patients with available HoNOS data than desired: Firstly, the low number of HoNOS data is mostly caused by the network’s short period of operating. Networks that started their services in 2012 did not have a large number of patients who were enrolled long enough to have to complete HoNOS data both for t0 and t1 (after 6 months). Secondly, about half the patients that were treated long enough to have their data considered in the study could in fact be included. Most time a delay in data transfer is assumed as the reason for missing data. But also the high number of incomplete filled HoNOS questionnaires points to that networks – despite being contractually obliged to assess HoNOS – were not well prepared to do so. The decision to assess HoNOS routinely was taken by the TK long before this research project was even thought of. However, it shows that only obliging service providers to fill an assessment tool without an evaluation plan and feedback system produces poor assessment compliance. However, these technical problems with HoNOS collections seem not to have caused a considerable bias, since included patients do not differ from excluded patients and the study population can still be regarded as representative.

The fact that the networks were similar on the central and standardised components of care and differed only slightly in structures and processes beyond central components, may explain why multivariate analysis was not successful in explaining differences in outcome between the networks. In addition, the observation period of only 6 months was probably too short for factors less prominent than the mere fact of providing home treatment.

Our findings need to be interpreted by also considering the fact that the applied method only presents correlations but no causal relationship. Finally, we analysed the data at patient level by disaggregating the network level information. This might have biased our results. It would have been better if we could have used more information related to the individual patient, such as how many hours of face-to-face contact were received. However, such data was not available. A further problem relates to structures and processes in the networks being measured by self-measurement of the professionals. While items such as “number of patients per case manager” relies on clear data that is available in the administration of the networks, item such as “average number of hours of face-to-face contacts with patients during the last month” should be based on the own documentation of the case managers and thus might be subject to bias.

## Conclusion

The home treatment networks observed in this study succeed in providing a higher intensity of care, including family intervention to the more psychosocially impaired patients and real life routine conditions. The fact that home treatment is provided at all seems to be more important in the first 6 months of treatment than details of what treatment was provided. However, there are signs indicating that being treated in a network that provides sociotherapy, psychoeducation, and provides patients with a treatment plan might be of some relevance to patient outcome. These findings might become more evident if treatment were observed for a longer follow-up period. Future research should consider this. Finally, this research suggests that for improving home treatment networks, it is advisable to invest in staff and employ highly experienced staff who are satisfied with their pay and who are ready to put a lot of effort into their work.

## Abbreviations

FACT, Functional Assertive Community Treatment; HoNOS, Health of the Nation Outcome Scales; NWpG, NetzWerk psychische Gesundheit (German Network for Mental Health); TK, Techniker Krankenkasse; WINEG, Wissenschaftliches Institut der Techniker Krankenkasse für Nutzen und Effizienz im Gesundheitswesen (Scientific Institute of TK for Benefit and Efficiency in Health Care).

## Additional files

Additional file 1: Network questionnaire on Integrated Care (IC). (PDF 193 kb) Additional file 2: Staff questionnaire on Integrated Care (IC). (PDF 141 kb)
