# Supplementary material for: Structures and processes necessary for providing effective home treatment to severely mentally ill persons: a naturalistic study
Source: BMC Psychiatry. 2016 Jul 15;16:242. doi: 10.1186/s12888-016-0945-z (PMC4946100; doi:10.1186/s12888-016-0945-z)
Supplement: Additional file 1: — Network questionnaire on Integrated Care (IC). (PDF 193 kb) [file 12888_2016_945_MOESM1_ESM.pdf]

Institute for Applied Quality Improvement and
Research in Health Care GmbH

NETWORK QUESTIONNAIRE ON INTEGRATED CARE (IC)

General information

1. NWpG contract partner: ______________________________
2. Provider of the NWpG contract partner: ______________________________
3. NWpG since: ______________ (month/year)
4. Characteristics of the region *(multiple responses allowed)*:
   - ❑ urban
   - ❑ provincial
   - ❑ rural
5. Number of service sites: ________
6. Is a care concept available? ❑ yes ❑ no *(→ continue with question 8)*
7. If yes, please name the concept: ______________________________
8. Regarding integrated care (IC), are case managers[1] working in “tandem” (i.e., patient contacts take place with at least two case managers)? ❑ yes ❑ no ❑ only if required
9. Are there regular meetings with cooperation partners to discuss the development of general structures and processes of IC (e.g. meetings with network management and representatives from cooperating providers)? ❑ yes ❑ no *(→ continue with question 11)*
10. If yes, frequency of these meetings:
    - ❑ never
    - ❑ twice a week
    - ❑ weekly
    - ❑ monthly
    - ❑ once in three months
    - ❑ once in six months
    - ❑ once a year
    - ❑ only if required
11. What is the provider’s motivation to offer IC? *(multiple responses allowed)*
    - ❑ new business segment
    - ❑ appropriate to the providers concept
    - ❑ financial incentives
    - ❑ market niche
    - ❑ ideal of mental healthcare

    Other reasons *(please list)*

    ____

[1] Definition of case manager: A case manager is a team member of the network, who is responsible for one or more patients, i.e. is the contact person, is responsible for care and provides care to the patient.

© 2013 AQUA-Institute GmbH

12. What are the strengths of your network?

____

____

____

____

____

Capacity in IC

The following questions refer to all patients in integrated care, regardless of their health insurance company.

13. Number of all enrolled patients treated in IC, regardless of their health insurance company on Nov. 1, 2012: __________ (number of patients)

14. Number of all enrolled patients treated in IC, regardless of their health insurance company on Oct. 31, 2013: __________ (number of patients)

15. Number of all enrolled patients treated in IC, regardless of their health insurance company, with a psychiatric or psychosomatic inpatient stay between Nov. 1, 2012 – Oct. 31, 2013: __________ (number of patients)

16. Number of all enrolled patients treated in IC, regardless of their health insurance company, with a case manager on Oct. 31, 2013: __________ (number of patients)

The following questions refer to all patients in integrated care, who are insured with the Techniker Krankenkasse (TK patients).

Depending on the effort, you may estimate the answers for the following questions.

17. Number of TK patients enrolled in IC on Nov. 1, 2012: __________ (number of TK patients)

18. Number of TK patients enrolled in IC on Oct. 31, 2013: __________ (number of TK patients)

19. Number of TK patients who received counseling regarding IC between Nov. 1, 2012 – Oct. 31, 2013: __________ (number of TK patients)

20. Number of TK patients newly enrolled between Nov. 1, 2012 – Oct. 31, 2013: __________ (number of TK patients)

© 2013 AQUA-Institute GmbH

21. Number of TK patients, where enrollment was declined by NWpG between Nov. 1, 2012 – Oct. 31, 2013: ____________ (number of TK patients)

22. Number of enrolled TK patients, where the network terminated care ahead of time (e.g. because of lack of compliance by TK patient) between Nov. 1, 2012 – Oct. 31, 2013: ____________ (number of TK patients)

23. Number of enrolled TK patients, where care was terminated ahead of time for other reasons (e.g. moving, death) between Nov. 1, 2012 – Oct. 31, 2013: ____________ (number of TK patients)

24. Number of enrolled TK patients, who terminated care as scheduled between Nov. 1, 2012 – Oct. 31, 2013: ____________ (number of TK patients)

Origin and provider of [name of NWpG contract partner]

What is the origin of [name of NWpG contract partner]?

- ❑ different community mental health services
- ❑ psychiatric hospital/department
- ❑ outpatient psychiatrist
- ❑ psychiatric home care
- ❑ day clinic
- ❑ day care center
- ❑ psychiatric outpatient clinic
- ❑ sociopsychiatric service
- ❑ assisted living
- ❑ therapeutic residential groups
- ❑ residential home
- ❑ counseling center
- ❑ sociotherapy
- ❑ work rehabilitation service

Others *(please list)*

____

- ❑ newly founded

25. Is [name of NWpG contract partner] solely a management company or are case managers employed also?

- ❑ solely management company
- ❑ management company with case managers

© 2013 AQUA-Institute GmbH

26. What other kind of services, apart from integrated care, are provided? If [name of NWpG contract partner] is solely a management company without case managers what other kind of services are provided by the contract partners apart from integrated care? *(multiple responses allowed)*

- ❑ Psychiatric hospital/department
- ❑ Psychosomatic hospital/department
- ❑ Outpatient psychiatrists
- ❑ Psychatric home care
- ❑ Day clinic
- ❑ Day care center
- ❑ Psychiatric outpatient clinic
- ❑ Sociopsychiatric service
- ❑ Assisted living
- ❑ Therapeutic residential groups
- ❑ Residential home
- ❑ Counseling center
- ❑ Sociotherapy
- ❑ Ergotherapy
- ❑ Work rehabilitation service
- ❑ Social workers
- ❑ None

Other *(please list)*

____

27. Number of employees at [name of NWpG contract partner] in full time equivalents. If there is no contract partner, please state “not applicable”. ____________ (number of full time equivalents)

28. Number of employees at [name of NWpG contract partner], who are responsible for IC management (employees not responsible for patient care e.g. secretary, controlling, quality management) in full time equivalents. ____________ (number of full time equivalents)

Case managers

29. Number of case managers in full time equivalents: ____________ (number of full time equivalents)

30. Number of case managers in full time equivalents for following professions:

   a. Psychologist with further training (officially recognized) in mental healthcare: ____________ (number of full time equivalents)

   b. Psychologist without further training in mental healthcare: ____________ (number of full time equivalents)

   c. Social worker with further training (officially recognized) in mental healthcare: ____________ (number of full time equivalents)

   d. Social worker without further training in mental healthcare: ____________ (number of full time equivalents)

   e. Ergotherapist with further training (officially recognized) in mental healthcare: ____________ (number of full time equivalents)

   f. Ergotherapist without further training in mental healthcare: ____________ (number of full time equivalents)

   g. Psychiatric nurse: ____________ (number of full time equivalents)

© 2013 AQUA-Institute GmbH

h. Nurse: ____________ (number of full time equivalents)

i. Peers with Ex-In training: ____________ (number of full time equivalents)

j. Peers without Ex-In training: ____________ (number of full time equivalents)

Others *(please list)*

k. ______________________________ ____________ (number of full time equivalents)

l. ______________________________ ____________ (number of full time equivalents)

m. ______________________________ ____________ (number of full time equivalents)

31. Is coordination for case management present? ❑ yes ❑ no *(→ continue with question 33)*

32. Number of employees in coordinating case management in IC in full time equivalents: ____________ (number of full time equivalents)

Team meetings

In a team meeting all available team members coordinate their work. A team meeting is normally a planned meeting.

33. Team meeting frequency:

❑ more than once a week
❑ weekly
❑ every second week
❑ monthly
❑ once in three months
❑ never
❑ only if required

Case meetings

A case meeting is defined as a meeting of two or more professionals involved in the care of a single patient to evaluate or plan care for this patient. Case meetings are normally planned meetings taking place without the patient.

Depending on the effort, you may estimate the answers for the following questions.

34. Are case meetings held as separate meetings (not as part of other meetings)? ❑ yes ❑ no ❑ only if required

35. Amount of hours for all case meetings for all patients, regardless of their health insurance company between Nov. 1, 2012 – Oct. 31, 2013: ____________ (sum of all hours)

36. Number of all patients, regardless of their health insurance company, subject of case meetings between Nov. 1, 2012 – Oct. 31, 2013: *(Patients subject of several case meetings count as one.)* ____________ (number of patients)

© 2013 AQUA-Institute GmbH

37. Average number of patients normally discussed in a one hour case meeting: ____________ (number of patients)

38. Participants in case meetings for patients, regardless of their health insurance company:

	always	often	seldom	never
a. Case manager				
b. Team management or coordinating case management				
c. Physician responsible for mental healthcare				
d. Network psychiatrist *(if c and d are the same, only rate c)*				
e. Cooperation partners				
f. Others *(please state)* ________________________				
g. Others *(please state)* ________________________				

Network dialogues

A network dialogue is a meeting where the patient and at least one other person from his social network, next to the case manager, evaluate or plan mental healthcare. Network dialogues are normally planned meetings.

39. Participants in network dialogues for patients, regardless of their health insurance company:

	always	often	seldom	never
a. Case manager				
b. Patient				
c. Physician responsible for mental healthcare				
d. Network psychiatrist *(if c and d are the same, only rate c)*				
e. Other therapist				
f. Relatives				
g. Friends				
h. Neighbours				
i. Employer				
j. Others *(please list)* ________________________				
k. Others *(please list)* ________________________				
40. Does every participant receive a protocol of the network dialogue?				

Standards of care

41. Are patient pathways available in written form? (e.g. manual, clinical pathway)? ❑ yes ❑ no

42. How often are the following processes carried out <u>during the enrollment of a patient</u> into IC?

	always	often	seldom	never
a. Counseling to inform about IC				
b. Anamnesis/assessment				
c. Home visit				
d. Contact to relatives				
e. Network dialogue				
f. Create crisis/emergency plan				
g. Consultation with the physician responsible for mental healthcare by patient and case manager				
h. Others *(please list)* ______________________				
i. Others *(please list)* ______________________				
j. Others *(please list)* ______________________				

24-hour hotline/crisis intervention

Please only answer if a 24-hour hotline is available.

Depending on the effort, you may estimate the answers for the following questions.

43. <u>Number of hotline contacts</u> by TK patients between Nov. 1, 2012 – Oct. 31, 2013: __________ (number of calls)

44. <u>Number of TK patients</u> who called the 24-hour hotline between Nov. 1, 2012 – Oct. 31, 2013: __________ (number of TK patients)

45. <u>Number of crisis interventions</u> for TK patients between Nov. 1, 2012 – Oct. 31, 2013: __________ (number of interventions)

46. <u>Number of hours</u> TK patients used <u>crisis intervention houses</u> between Nov. 1, 2012 – Oct. 31, 2013: __________ (number of hours)

47. Number of TK patients who used crisis intervention houses between Nov. 1, 2012 – Oct. 31, 2013: __________ (number of TK patients)

48. Number of TK patients whose physician responsible for mental healthcare can be reached in the following ways: *(Please state the number of patients.)*
    a. Always __________ (number of TK patients)
    b. Spontaneously during opening hours __________ (number of TK patients)
    c. During planned appointments __________ (number of TK patients)
    d. Not reachable __________ (number of TK patients)

© 2013 AQUA-Institute GmbH

Cooperation partners

49. Please insert the number of providers you have a cooperation agreement with and state the agreed payments.

a. Outpatient psychiatrist ____________ (number)
- ❑ no payments
- ❑ payments for services rendered ____________ Euro
- ❑ lump sum per patient contact ____________ Euro
- ❑ lump sum per quarter and patient ____________ Euro
- ❑ lump sum per quarter ____________ Euro

other kinds of payment *(please list)*
- ❑ ______________________________ ____________ Euro

b. General physician ____________ (number)
- ❑ no payments
- ❑ payments for services rendered ____________ Euro
- ❑ lump sum per patient contact ____________ Euro
- ❑ lump sum per quarter and patient ____________ Euro
- ❑ lump sum per quarter ____________ Euro

other kind of payments *(please list)*
- ❑ ______________________________ ____________ Euro

c. Psychiatric home care ____________ (number)
- ❑ no payments
- ❑ payments for services rendered ____________ Euro
- ❑ lump sum per patient contact ____________ Euro
- ❑ lump sum per quarter and patient ____________ Euro
- ❑ lump sum per quarter ____________ Euro

other kind of payments *(please list)*
- ❑ ______________________________ ____________ Euro

d. Psychiatric outpatient clinic ____________ (number)
- ❑ no payments
- ❑ payments for services rendered ____________ Euro
- ❑ lump sum per patient contact ____________ Euro
- ❑ lump sum per quarter and patient ____________ Euro
- ❑ lump sum per quarter ____________ Euro

other kind of payments *(please list)*
- ❑ ______________________________ ____________ Euro

© 2013 AQUA-Institute GmbH

e. Sociotherapist ___________ (number)
- ❑ no payments
- ❑ payments for services rendered ___________ Euro
- ❑ lump sum per patient contact ___________ Euro
- ❑ lump sum per quarter and patient ___________ Euro
- ❑ lump sum per quarter ___________ Euro

other kind of payments *(please list)*
- ❑ ___________________________ ___________ Euro

f. Ergotherapist ___________ (number)
- ❑ no payments
- ❑ payments for services rendered ___________ Euro
- ❑ lump sum per patient contact ___________ Euro
- ❑ lump sum per quarter and patient ___________ Euro
- ❑ lump sum per quarter ___________ Euro

other kind of payments *(please list)*
- ❑ ___________________________ ___________ Euro

g. Psychotherapist ___________ (number)
- ❑ no payments
- ❑ payments for services rendered ___________ Euro
- ❑ lump sum per patient contact ___________ Euro
- ❑ lump sum per quarter and patient ___________ Euro
- ❑ lump sum per quarter ___________ Euro

other kind of payments *(please list)*
- ❑ ___________________________ ___________ Euro

h. Day clinic ___________ (number)
- ❑ no payments
- ❑ payments for services rendered ___________ Euro
- ❑ lump sum per patient contact ___________ Euro
- ❑ lump sum per quarter and patient ___________ Euro
- ❑ lump sum per quarter ___________ Euro

other kind of payments *(please list)*
- ❑ ___________________________ ___________ Euro

© 2013 AQUA-Institute GmbH

i. Psychiatric hospital/department ____________ (number)
- ❑ no payments
- ❑ payments for services rendered ____________ Euro
- ❑ lump sum per patient contact ____________ Euro
- ❑ lump sum per quarter and patient ____________ Euro
- ❑ lump sum per quarter ____________ Euro

other kind of payments *(please list)*
- ❑ ____________________ ____________ Euro

j. Psychosomatic hospital/department ____________ (number)
- ❑ no payments
- ❑ payments for services rendered ____________ Euro
- ❑ lump sum per patient contact ____________ Euro
- ❑ lump sum per quarter and patient ____________ Euro
- ❑ lump sum per quarter ____________ Euro

other kind of payments *(please list)*
- ❑ ____________________ ____________ Euro

k. Others *(please list)*

________________________________ ____________ (number)
- ❑ no payments
- ❑ payments for services rendered ____________ Euro
- ❑ lump sum per patient contact ____________ Euro
- ❑ lump sum per quarter and patient ____________ Euro
- ❑ lump sum per quarter ____________ Euro

other kind of payments *(please list)*
- ❑ ____________________ ____________ Euro

l. Others *(please list)*

________________________________ ____________ (number)
- ❑ no payments
- ❑ payments for services rendered ____________ Euro
- ❑ lump sum per patient contact ____________ Euro
- ❑ lump sum per quarter and patient ____________ Euro
- ❑ lump sum per quarter ____________ Euro

other kind of payments *(please list)*
- ❑ ____________________ ____________ Euro

© 2013 AQUA-Institute GmbH

50. Number of providers in your region, where **no** cooperation agreements for IC exist.

Depending on the effort, you may estimate the answers for the following questions.

a. Outpatient psychiatrist ____________ (number)

b. Psychiatric home care ____________ (number)

c. Psychiatric outpatient clinic ____________ (number)

d. Sociotherapist ____________ (number)

e. Ergotherapist ____________ (number)

f. Psychotherapist ____________ (number)

g. Day clinic ____________ (number)

h. Psychiatric hospital/department ____________ (number)

i. Psychosomatic hospital/department ____________ (number)

Cooperations without official agreement

51. How often did you approximately cooperate with the following providers from Nov. 1, 2012 – Oct. 31, 2013 without any official agreement?

	always	often	seldom	never
a. Outpatient psychiatrist				
b. Psychiatric outpatient clinic				
c. General practitioner				
d. Psychiatric hospitel/department				
e. Psychosomatic hospital/department				
f. Day clinic				
g. Day care center				
h. Psychiatric home care				
i. Psychotherapist				
j. Sociotherapist				
k. Ergotherapist				
l. Physiotherapist				
m. Home care				
n. Social worker				
o. Household assistant				
p. Sociopsychiatric service				
q. Assisted living				
r. Residential home				
s. Counseling center				
t. Work rehabilitation service				
u. Integration service				
v. Self-help groups				
w. Job center				
x. Patients' employer				

© 2013 AQUA-Institute GmbH

	always	often	seldom	never
y. Training center				
z. Honorary service				
aa. Club, church, similar services (e.g. sports club, language course)				
bb. Others *(please list)* ______________________________				
cc. Others *(please list)* ______________________________				
dd. Others *(please list)* ______________________________				

Patient contacts

Depending on the effort, you may estimate the answers for the following questions.

52. Number of contacts with TK patients between Nov. 1, 2012 – Oct. 31, 2013 *(including face-to-face-contacts, telephone, E-Mail etc., no group activities):* __________ (number of contacts)

53. Is there a minimum contact frequency for TK patients (e.g. each patient should be contacted at least once in three months)?
    - ❑ yes
    - ❑ no (→ *continue with question 55*)
    - ❑ only if required (→ *continue with question 55*)

54. Minimum contact frequency for TK patients:
    - ❑ weekly
    - ❑ once in two weeks
    - ❑ monthly
    - ❑ once in three months
    - ❑ once in six months

55. Number of face-to-face contacts with TK patients between Nov. 1, 2012 – Oct. 31, 2013 *(no group activities):* __________ (number of face-to-face-contacts)

56. Number of home visits for TK patients between Nov. 1, 2012 – Oct. 31, 2013: __________ (number of visits at home)

57. Number of contacts with TK patients' social network/relatives between Nov. 1, 2012 – Oct. 31, 2013: __________ (number of contacts)

58. Number of hours TK patients participated in group activities between Nov. 1, 2012 – Oct. 31, 2013: __________ (number of hours)

© 2013 AQUA-Institute GmbH

Services for relatives

Services for relatives are structured services within IC, focusing on relatives and not on patients. (No network dialogues.)

59. What kind of services for relatives does the network provide? *(multiple responses allowed)*

- ❑ Psychoeducation
- ❑ Relative groups/self-help-groups/inclusion groups
- ❑ Conversations for relatives
- ❑ Counseling for relatives

Others *(please list)*

____

- ❑ No services for relatives

Group activities for patients

60. What kind of group activities for patients does the network provide? *(multiple responses allowed)*

- ❑ Psychoeducation groups
- ❑ Crisis planning groups
- ❑ Sports /relaxation groups

Others *(please list)*

____

____

- ❑ No group activities for patients

Documentation

	always	often	seldom	never
61. Does the documentation include the kind of service that was provided?				
62. Does the documentation include who provided the service?				
63. Does the documentation include when the service was provided?				
64. Does the documentation include the duration of service provision?				

© 2013 AQUA-Institute GmbH

65. Who can access the documentation?

	whole	partial	No access
a. Case manager:			
b. Network's physician responsible for mental healthcare:			
c. External physician responsible for mental healthcare:			
d. Crisis teams:			
e. Patient:			
f. All cooperation partners:			
Others: *(please list)*<br>g. ______________________			
h. ______________________			
i. ______________________			

Evaluation

66. Do you evaluate your network? ❑ yes ❑ no *(→ continue with question 68)*

67. How do you evaluate your network?

____

____

68. Does a quality management system exist? ❑ yes ❑ no

69. Does the network participate in other research projects? ❑ yes ❑ no *(→continue with question 71)*

70. In which other research projects does the network participate?

____

____

71. Are there any suggestions to improve the NWpG?

____

____

____

____

____

Thank you!

© 2013 AQUA-Institute GmbH
