# Supplementary material for: Structures and processes necessary for providing effective home treatment to severely mentally ill persons: a naturalistic study
Source: BMC Psychiatry. 2016 Jul 15;16:242. doi: 10.1186/s12888-016-0945-z (PMC4946100; doi:10.1186/s12888-016-0945-z)
Supplement: Additional file 2: — Staff questionnaire on Integrated Care (IC). (PDF 141 kb) [file 12888_2016_945_MOESM2_ESM.pdf]

Institute for Applied Quality Improvement and
Research in Health Care GmbH

STAFF QUESTIONNAIRE ON INTEGRATED CARE (IC)

General information

1. Age: ______ years
2. Sex: ❑ female
   ❑ male
3. Profession: *(e.g. nurse, ergotherapist etc.)*

   ____

4. Advanced training: *(e.g. psychiatric nurse, sociotherapy, Ex-In-training etc.)*

   ____

5. Number of total weekly working hours ____________ weekly working hours
6. Estimated number of weekly working hours in integrated care (IC) *(not all colleagues are solely working in IC)* ____________ weekly working hours
7. What is your gross salary for one year? ____________ Euro
8. How long have you already been working in IC? _______/_____ years/months
9. In general, how long have you been working in mental healthcare? ____________ years
10. What is your motivation to work in IC? *(multiple responses allowed)*

    ❑ My employer provides IC.
    ❑ In IC I can work independently.
    ❑ In IC I have more flexible working hours.
    ❑ In IC I can realize my ideals/visions of mental healthcare.

    Other reasons *(please list)*

    ____

11. What is a burden of work in IC for you?

    ____

    ____

12. What enriches your work in IC?

    ____

    ____

© 2013 AQUA-Institute GmbH

Contacts to patients insured with Techniker Krankenkasse (TK patients) in November 2013

The following information should refer to the time period from November 1st to 30th, 2013. If you were absent for more than five days in November 2013 due to vacation or illness, please provide information from the last month in which you were absent for five or less days. For "month" you can either choose a calendar month or a similar time period, e.g. October 10th to November 9th, 2013. It is important that you can fairly well remember the time period in question.

The following questions focus exclusively on TK patients enrolled in IC (TK patients). Depending on the effort, you may estimate the answers for the following questions.

13. a) How many TK patients did you see face-to-face during the month in question?	___________	number of TK patients
b) How many face-to-face contacts to TK patients did you have in the month in question? *(Please state the number of contacts.)*	___________	number of face-to-face contacts
c) Duration of all face-to-face contacts to TK patients within the month in question: *(Please state the total duration in hours.)*	___________	number of hours of face-to-face contact
14. a) How many TK patients did you see in home treatment during the month in question? *(Please state the number of TK patients.)*	___________	number of TK patients
b) How many home visits did you do at TK patients' homes during the month in question?	___________	number of home visits
c) Duration of all home visits at TK patients' homes within the month in question: *(Please state the total duration in hours.)*	___________	number of hours of home visits
15. a) How many TK patients did you contact via e-mail, telephone or mail during the month in question? *(Please state the number of TK patients.)*	___________	number of TK patients
b) How many TK patients did you exclusively contact via e-mail, telephone or mail during the month in question? *(Please state the number of TK patients.)*	___________	number of TK patients
16. How many TK patients did you see relatives from during the month in question? *(Please state the number of TK patients.)*	___________	number of TK patients
17. How many TK patients were assigned to you in the month[1] in question?	___________	number of TK patients
18. How many TK patients assigned to you did you not contact, neither face-to-face nor e-mail, telephone or mail during the month in question? *(Please insert the number of TK-patients.)*	___________	number of TK patients
19. How many TK patients assigned to you did you talk about with the physician responsible for their mental healthcare during the month in question?	___________	number of TK patients

[1] Patients who were assigned to you are the patients you are responsible for (e.g. you are the case manager of these patients).

© 2013 AQUA-Institute GmbH

(Please state the number of TK patients.)		
20. Of how many enrolled TK patients <u>assigned to you,</u> can reach the physician responsible for their mental healthcare in the following ways:<br>*(Please state the number of TK patients.)*		
a. Always	____________	number of TK patients
b. Spontaneously during opening hours	____________	number of TK patients
c. During planned appointments	____________	number of TK patients
d. Not reachable	____________	number of TK patients

Cooperations

21. How often did you approximately cooperate with the following services or providers during the **twelve months starting from December 1st, 2012 to November 30th, 2013?**

	usually always	often	seldom	never	not available
a. Outpatient psychiatrist					
b. Psychiatric outpatient clinic					
c. General practitioner					
d. Psychiatric hospital/department					
e. Day clinic					
f. Day care center					
g. Psychiatric home care					
h. Psychotherapist					
i. Sociotherapist					
j. Ergotherapist					
k. Physiotherapist					
l. Home care					
m. Social worker					
n. Household assistant					
o. Sociopsychiatric service					
p. Assisted living					
q. Residential home					
r. Counseling center					
s. Work rehabilitation service					
t. Integration service					
u. Job center					
v. Patients' employer					
w. Training center					
x. Honorary service					
y. Club, church, similar services (e.g. sports club, language course, choir)					
z. Self-help groups					

© 2013 AQUA-Institute GmbH

	usually always	often	seldom	never	not available
Others *(please list)* aa.____________________					
Others *(please list)* bb.____________________					
Others *(please list)* cc.____________________					

22. **As of November 30th, 2013:** For how many TK patients assigned to you could you arrange the following services or providers? *(Services or providers that were already used by the patient before enrollment should not be considered here.)*

a. Outpatient psychiatrist: __________ number of TK patients
b. Psychiatric outpatient clinic: __________ number of TK patients
c. General practitioner: __________ number of TK patients
d. Psychiatric hospital/department: __________ number of TK patients
e. Day clinic: __________ number of TK patients
f. Day care center: __________ number of TK patients
g. Psychiatric home care: __________ number of TK patients
h. Psychotherapist: __________ number of TK patients
i. Sociotherapist: __________ number of TK patients
j. Ergotherapist: __________ number of TK patients
k. Physiotherapist: __________ number of TK patients
l. Home care: __________ number of TK patients
m. Sociopsychiatric service: __________ number of TK patients
n. Household assistant: __________ number of TK patients
o. Assisted living: __________ number of TK patients
p. Residential home: __________ number of TK patients
q. Counseling center: __________ number of TK patients
r. Work rehabilitation service: __________ number of TK patients
s. Integration service: __________ number of TK patients
t. Self-help groups: __________ number of TK patients
u. Social worker: __________ number of TK patients
v. Job center: __________ number of TK patients
w. Payed work: __________ number of TK patients
x. Training: __________ number of TK patients

© 2013 AQUA-Institute GmbH

y. Internship: ______________ number of TK patients

z. In honorary services: ______________ number of TK patients

aa. Club, church, similar services (e.g. sports club, language course): ______________ number of TK patients

Others *(please list)*
bb. ____ ______________ number of TK patients

Others *(please list)*
cc. ____ ______________ number of TK patients

Others *(please list)*
dd. ____ ______________ number of TK patients

Services

Depending on the effort, you may estimate the answers for the following questions.

23. How many TK patients assigned to you received the following services from **July 1st to September 30th, 2013**? The services named need not have been provided solely by yourself. *(If you do not provide a service, please insert 99.)*

a. Psychoeducation: ______________ number of TK patients

b. Intensive care: ______________ number of TK patients

c. Stabilization: ______________ number of TK patients

d. Crisis intervention (ambulatory): ______________ number of TK patients

e. Psychiatric home care: ______________ number of TK patients

f. Home treatment *(only to be answered if different from psychiatric home care)*: ______________ number of TK patients

g. Sociotherapy: ______________ number of TK patients

h. Psychotherapy: ______________ number of TK patients

i. Case meetings: ______________ number of TK patients

j. Network dialogues[2]: ______________ number of TK patients

k. Counseling for relatives[3]: ______________ number of TK patients

l. Contact to patients' general physician: ______________ number of TK patients

m. Contact to patients' physician responsible for mental healthcare: ______________ number of TK patients

n. Contact to patients' psychotherapist: ______________ number of TK patients

[2] Meetings, where the patient and at least one other person from his social network, next to the case manager, evaluate or plan mental healthcare. Network dialogues are normally planned meetings.
[3] Counseling for relatives is a structured service within IC, focusing on relatives and not on patients (presence of patient not necessary).

© 2013 AQUA-Institute GmbH

Others *(please list)*
o. ______________________________ ____________ number of TK-patients

Others *(please list)*
p. ______________________________ ____________ number of TK-patients

24. How many TK patients assigned to you received a copy of their treatment plan? ____________ number of TK-patients

25. How many TK patients assigned to you have been enrolled in IC for three months or longer? ____________ number of TK-patients

26. How many TK patients assigned to you received a copy of their crisis plan within the first three months after enrollment in IC? ____________ number of TK-patients

Distribution of work tasks

27. Referring to a “typical week”, please estimate the amount of working time you spend on the following tasks. Please include all tasks for enrolled patients, regardless of whether the patient is assigned to you or insured with the TK. *(Please do not extend 100 %.)*

a.	Face-to-face contact with patients	____________	%
b.	Other contact with patients (e.g. telephone, e-mail)	____________	%
c.	Coordination of care (without patient contact) including case or team meetings, supervision	____________	%
d.	Services for relatives/network dialogues	____________	%
e.	Documentation and administration	____________	%
f.	Driving for home visits	____________	%
	Others *(please list)*		
g.	______________________________	____________	%
	Others *(please list)*		
h.	______________________________	____________	%
**Sum**		100	%

© 2013 AQUA-Institute GmbH

Supervision and advanced training

The following questions cover the **last twelve months from December 1st, 2012 to November 30th, 2013.**

28. How many hours of supervision did you receive in the last twelve months? ____________ number of hours

29. Who carried out this supervision? *(multiple responses allowed)*

❑ Superior
❑ External
❑ Psychiatrist (internal)

Others (*please list*)

____

30. How many hours of advanced training did you receive in the last twelve months? ____________ number of hours

a. How many hours of training on systemic care did you receive in the last twelve months? ____________ number of hours

b. How many hours of training on Need Adapted Treatment did you receive in the last twelve months? ____________ number of hours

c. How many hours of training on other care concepts did you receive in the last twelve months (e.g. care concept of IVPNetworks)?

Please state ______________________ ____________ number of hours

Please state ______________________ ____________ number of hours

31. Your suggestions for improving IC of TK:

____

____

____

____

____

© 2013 AQUA-Institute GmbH

32. With the following questions we would like to know more about your working conditions and job satisfaction. We make use of questions from validated and well-established questionnaires used regularly for surveys. Please mark (with a cross) to what extent you agree with the following statements on your working conditions.

	strongly disagree	disagree	agree	strongly agree
a. I have constant time pressure due to a heavy work load.				
b. I have many interruptions and disturbances while performing my job.				
c. Over the past few years, my job has become more and more demanding.				
d. I receive the respect I deserve from my superior or a respective relevant person.				
e. My job promotion prospects are poor.				
f. I have experienced or I expect to experience an undesirable change in my work situation.				
g. My job security is poor.				
h. Considering all my efforts and achievements, I receive the respect and prestige I deserve at work.				
i. Considering all my efforts and achievements, my job promotion prospects are adequate.				
j. Considering all my efforts and achievements, my salary/income is adequate.				
k. I get easily overwhelmed by time pressures at work.				
l. As soon as I get up in the morning I start thinking about work problems.				
m. When I get home, I can easily relax and 'switch off' work.				
n. People close to me say I sacrifice too much for my job.				
o. Work rarely lets me go, it is still on my mind when I go to bed.				
p. If I postpone something that I am supposed to do today, I'll have trouble sleeping at night.				

© 2013 AQUA-Institute GmbH

33. Please mark to what extent you are satisfied with the following working conditions. (scale 1 – 7, with: 1 = strongly dissatisfied; 7 = strongly satisfied).

	strongly dissatisfied						strongly satisfied
	1	2	3	4	5	6	7
a. The physical work conditions							
b. The freedom to choose your own method of working							
c. Your fellow workers							
d. The recognition you get for good work							
e. The amount of responsibility you are given							
f. Your rate of pay							
g. Your opportunity to use your abilities							
h. Your job hours							
i. The amount of variety in your job							
j. Now, taking everything into consideration, how do you feel about your job as a whole?							

Thank you!

© 2013 AQUA-Institute GmbH
